# The Operation of Cæsarean Section Performed on a Cow

**Published:** 1887-07

**Authors:** C. Hamilton

**Affiliations:** Ringgold, Georgia


### THE OPERATION OF CÆSAREAN SECTION PERFORMED SUCCESSFULLY ON A COW.

By C. HAMILTON, M. D., OF RINGGOLD, GA.

On the 20th day of April I was called some distance in the country to see a patient. On my arrival I was asked by the gentleman of the house to come out to the lot as soon as I had made a prescription for his daughter, whom I had visited. In the lot I found a cow in labor, and had been for about twenty-four hours. On examination I found that, owing to an injury that she had received on the railroad, there was not sufficient passage for the calf. Its fore feet were protruding, and there was no room for the passage of its head. The gentleman insisted on saving the life of the calf; so I concluded to perform the Cæsarean section, though I had never heard of this operation having been performed on a cow; so, without the use of any anæsthetic, I immediately began the operation by making an incision a little to the right of the median line, commencing just at the edge of the mammary gland, and cutting up made an incision six or seven inches long, and the cut in the uterus as small as would admit the calf. As the membranes had not ruptured, I waited a few minutes and then proceeded to rupture them and deliver the calf. In a few minutes it was up, walking about. I used a catgut suture in closing the cut in the uterus. A few minutes after the cuts were closed the cow got up and let the calf suck. There was very little swelling or suppuration during the healing of the wound. The lochial discharge commenced at once and continued its usual time. I directed that she should be fed on meal and bran for fifteen or twenty days.

I learn that she has been giving sufficient milk for the calf all the time, and at this date is doing well, running in the range with other cattle. It is strange to say that the protrusion of the membranes and the contraction of the uterus at the cut was just the same as at the mouth, showing very plainly that the contractility of the uterus is in every part and direction; while the uterus was cut laterally, the contraction was great and the pains as regular as in natural labor. The cow struggled but little during the operation, and what little she did seemed to assist in delivery.

